# Thermally-Responsive Loading and Release of Elastin-Like Polypeptides from Contact Lenses

**DOI:** 10.3390/pharmaceutics11050221

**Published:** 2019-05-07

**Authors:** Wan Wang, Changrim Lee, Martha Pastuszka, Gordon W. Laurie, J. Andrew MacKay

**Affiliations:** 1 Department of Pharmacology and Pharmaceutical Sciences, School of Pharmacy, University of Southern California, Los Angeles, CA 90033, USA; leilawangwan@gmail.com (W.W.); changril@usc.edu (C.L.); martha.past@gmail.com (M.P.); 2 Department of Cell Biology, School of Medicine, University of Virginia, Charlottesville, VA 22908, USA; gwl6s@virginia.edu; 3 Department of Biomedical Engineering, Viterbi School of Engineering, University of Southern California, Los Angeles, CA 90089, USA; 4 Department of Ophthalmology, USC Roski Eye Institute, Keck School of Medicine, University of Southern California, Los Angeles, CA 90089, USA

**Keywords:** elastin-like polypeptide (ELPs), contact lens, lacritin, protein therapeutics, drug delivery

## Abstract

Contact lenses are widely prescribed for vision correction, and as such they are an attractive platform for drug delivery to the anterior segment of the eye. This manuscript explores a novel strategy to drive the reversible adsorption of peptide-based therapeutics using commercially available contact lenses. To accomplish this, thermo-sensitive elastin-like polypeptides (ELPs) alone or tagged with a candidate ocular therapeutic were characterized. For the first time, this manuscript demonstrates that Proclear Compatibles^TM^ contact lenses are a suitable platform for ELP adsorption. Two rhodamine-labelled ELPs, V96 (thermo-sensitive) and S96 (thermo-insensitive), were employed to test temperature-dependent association to the contact lenses. During long-term release into solution, ELP coacervation significantly modulated the release profile whereby more than 80% of loaded V96 retained with a terminal half-life of ~4 months, which was only 1–4 days under solubilizing conditions. A selected ocular therapeutic candidate lacritin-V96 fusion (LV96), either free or lens-bound LV96, was successfully transferred to HCE-T cells. These data suggest that ELPs may be useful to control loading or release from certain formulations of contact lenses and present a potential for this platform to deliver a biologically active peptide to the ocular surface via contact lenses.

## 1. Introduction

As growth factors and peptides derived from the tear proteome are explored as novel therapies for the anterior segment [1], it may be worthwhile to explore new drug delivery platforms that can be integrated with contact lenses [2]. New platforms may benefit from being biocompatible, biodegradable, and compatible with existing medical devices [3]. One such platform explored by our group and others are the elastin-like polypeptides (ELPs) [4]. ELPs are composed of repeated pentameric peptides, (Val-Pro-Gly-Xaa-Gly)*_n_*. They reversibly phase separate from aqueous solution above a transition temperature (*T_t_*) which can be tuned by adjusting the identity of a guest amino acid (Xaa) and the length (*n*) [5]. Like parent ELPs, ELP fusion proteins ‘coacervate’ above *T_t_*; furthermore, this assembly process can functionalize pharmacologically drug carriers [6] or imaging probes [7]. Our group previously demonstrated the encapsulation [8] and fusion [9] ability of thermo-responsive elastin-like polypeptides (ELPs) carrying either small molecules or protein treatments to ocular tissue. Now, this manuscript reports the surprising discovery that ELPs significantly attach and dramatically extend release from a commercially available contact lens, Proclear Compatibles^TM^. Using this discovery, two hypotheses were tested: 1) coacervation enhances attachment and slows the detachment of ELPs to and from contact lens; 2) ELP fusions with a biologically active peptide can transfer proteins from the lens to a cell-culture model of the corneal epithelium. By involving two types of ELPs, V96 (thermo-sensitive) and S96 (thermo-insensitive), the data show that the attachment and release of ELPs to contact lenses is both ELP and incubation temperature dependent. As a proof of the concept that ELPs can deliver a fusion protein, we modified the lens with a prosecretory mitogenic fusion called LV96 and demonstrated that the proximity between the LV96 on the contact lens enhances transfer to cultured human corneal epithelial cells.

## 2. Materials and Methods

### 2.1. Synthesis, Expression and Purification of ELPs

cDNAs encoding either ELPs V96, S96, or LV96 were cloned into the pET-25b(+) vector that was originally purchased from Novagen (#69753, Madison, WI, USA) and further modified for ELP or ELP fusion cloning [10]. The cloned constructs were sequenced, transformed into and expressed in BLR(DE3) competent *Escherichia* Coli (*E*. Coli) (#69053, Novagen, Madison, WI, USA). For V96 and S96, both were fermented in terrific broth media for 16–18 h at 37 °C without isopropyl β-D-1-thiogalactopyranoside (IPTG). For LV96, it was fermented in terrific broth media for 4 h at 37 °C followed by 0.5 mM IPTG induction. The temperature was immediately decreased to 25–30 °C and it was fermented for another 5–6 h. For all ELPs, the supernatant was subjected to ELP-mediated phase separation in 2 M sodium chloride at 37 °C after bacterial cell lysis and clarification of cell debris by centrifugation. Coacervates were immediately pelleted after the phase separation was observed (hot-spin). After centrifugation, soluble impurities (supernatant) were removed and coacervates (pellet) were resolubilized in clean ice-cold phosphate buffered saline (PBS). Thoroughly resolubilized ELPs were centrifuged to remove any insoluble impurities (cold-spin). After the cold-spin, the supernatant was transferred to a clean tube. Cycles of hot-spin followed by cold-spin were repeated 3 times to achieve the necessary purity. LV96 was further subjected to size exclusion chromatography to remove the cleaved byproduct.

### 2.2. ELPs Inverse Phase Transition Characterization

The *T_t_*-concentration phase diagrams for rhodamine-labeled ELPs or ELP fusion proteins were characterized by optical density observation at 350 nm (OD 350nm) using a DU800 UV–Vis spectrophotometer (Beckman Coulter, CA, USA) as a function of solution temperature. Different concentrations of ELPs (5, 10, 25, 50, and 100 μM) were heated at 1 °C/min from 10 to 85 °C and OD 350 nm was recorded every 0.3 °C. *T_t_* was defined at the point of the maximum first derivative. The *T_t_* from each concentration was used to plot the phase diagram and fit with Equation 1.

(1) $$T_{t}=b-m\;\log_{10}\left[C_{ELP}\right]$$

### 2.3. Rhodamine Labeling of V96, S96 and LV96, and Decoration of Proclear Compatibles^TM^ Contact Lenses

ELPs were covalently modified with N-hydroxysuccinimide (NHS)-Rhodamine (Thermo Fisher Scientific Inc, Rockford, IL, USA). The conjugation was performed in 100 mM borate buffer (pH 8.5) overnight at 4 °C to covalently conjugate amine reactive NHS-esters to the primary amine at the ELP amino terminus. Excess fluorophore was removed using a desalting PD-10 column (GE Healthcare, Piscataway, NJ, USA) and overnight dialysis against PBS at 4 °C. For the initial screening study, contact lenses were either incubated with 50 µM labeled ELPs overnight at 37 °C in a 24-well plate or spot-decorated with concentrated, labeled ELPs using a 20-µL pipette at 37 °C. Proclear Compatibles^TM^ contact lenses (CooperVision, Inc., Lake Forest, CA, USA) were incubated in 100 µM rhodamine-labeled V96 or S96 for 48 h at 4 or 37 °C. After a gentle rinse with ddH_2_O at 4 or 37 °C, contact lenses were immediately imaged using Zeiss 510 confocal laser scanning microscopy (Carl Zeiss AG, Oberkochen, Germany), respectively at 37 or at 4 °C, and quantified using ImageJ. Decoration of LV96 onto contact lenses in a ring shape was achieved by overnight incubation of 50 µM rhodamine-labeled LV96 with Proclear Compatibles^TM^ contact lens at 37 °C followed by washing off LV96 attached at the center of the lens using ice-cold PBS and pipetting out.

### 2.4. Characterization of Release Kinetics of ELPs from Proclear Compatibles^TM^ Contact Lenses

Contact lenses were incubated in 100 µM rhodamine-labeled V96 or S96 for 24 h at 4 °C or 37 °C. After one gentle rinse with PBS at 4 or 37 °C, contact lenses were immediately placed into 4 mL of PBS at 4 or 37 °C for 1 week. Small aliquots of the solution (100 µL) were withdrawn at predetermined intervals (5, 15, 30 min, 1, 2, 4, 8, 24, 48, 72, 96, 120, 168 h) and kept at −20 °C. After one week, lenses were thoroughly washed in PBS at 4 °C for 24 h to detach ELPs. Fluorescence intensity of collected samples was measured spectrophotometrically (Ex: 525 nm, Em: 575 nm) using a Synergy™ H1m Monochromator-Based Multi-Mode Microplate Reader (BioTek Instruments, Inc., Winooski, VT, USA) and analyzed using built-in Gen5 2.01 Data Analysis Software (BioTek). Total fluorescence on the lens was calculated using Equation 2. Since the measurement of the contact lens-bound fraction at each time point was distorted due to the convex shape of the contact lenses, the percent of retention on the lens was defined at each time point using Equation 3. Using GraphPad Prism (Prism Software, Irvine, CA, USA), these retention data failed to fit one-phase dissociation model; however, a two-phase dissociation model (Equation 4) fit well to the observed profiles. Goodness of fit and predicted values are reported.

(2) $$Total\;I_{rhodamine}=I_{release\_Total}+I_{wash\_Total}$$

(3) $$Retention\left(t\right)=\frac{Total\;I_{rhodamine}-\sum_{t=0}^{t}I_{release\_t}}{Total\;I_{rhodamine}}\times 100\%$$

(4) $$Retention\left(t\right)=Percent_{fast}\;e^{-k_{fast}t}+\left(100-Percent_{fast}\right)e^{-k_{slow}t}$$

(5) $$AUC_{0-Infinity}=AUC_{0-168h}\;+{\%}_{last}/k_{slow}$$

### 2.5. Human Corneal Epithelial Cells-Transformed with SV40 (HCE-T) Uptake Study

HCE-T cellular uptake was conducted on 35-mm glass coverslip-bottomed dishes. Briefly, HCE-T cells were grown to 70–80% confluence and gently rinsed with warm fresh medium before changing to fresh media containing either rhodamine-labeled lacritin (10 µM, protein concentration), LV96 (10 µM) or contact lenses loaded with rhodamine-labeled LV96. After incubation at 37 °C for 1 h, cells were rinsed with fresh media, incubated with 4′,6-diamidino-2-phenylindole (DAPI) for 15 min to stain nuclei, and then imaged using a Zeiss 510 confocal microscope system (Carl Zeiss AG, Oberkochen, Germany) with quantification by ImageJ (National Institutes of Health, Bethesda, MD, USA). To evaluate the transfer of contact lens-bound LV96 to the monolayer of HCE-T cells, images from different zones were directly obtained at the edge of the lens where the highest likelihood of direct contact between the lens and the monolayer occurred.

### 2.6. Statistical Analysis

All experiments were repeated at least three times. Statistical analysis was performed by Student’s *t*-test or one-way ANOVA followed by Tukey’s post-hoc test using statistical software IBM SPSS Statistics v21 (IBM Corp., Armonk, NY, USA). A *p* value of less than 0.05 was considered statistically significant.

## 3. Results

### 3.1. Expression and Purification of ELPs

**Figure 1:**
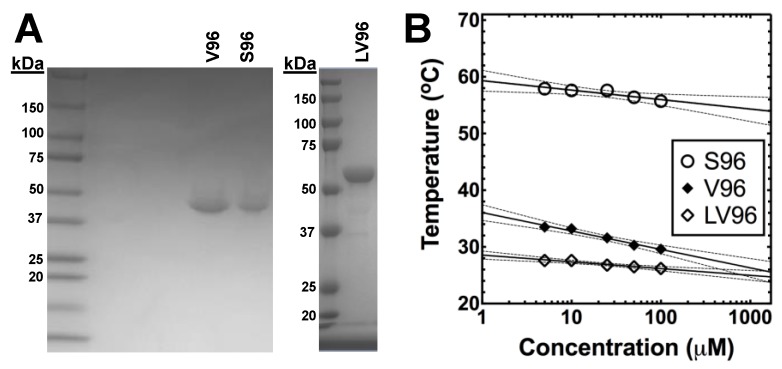
The purity, size, and temperature-dependent phase behavior of V96, S96, and LV96 evaluated for this study. (**A**) Identity and purity of V96, S96 and LV96 were analyzed by SDS-PAGE using Coomassie blue staining. (**B**) The phase transition temperature was plotted vs. concentration as a phase diagram, below which ELPs remains soluble, and fit with Equation 1. Solid line: Fit; Dashed line: 95% confidence interval of mean.

All ELPs involved in this study, V96, S96, and LV96, were heterologously expressed from a seamlessly cloned synthetic gene in *E. coli* (Table 1). Purification was done via inverse transition cycling [10], which is a non-chromatographic purification method that utilizes ELP-mediated phase separation from clarified bacterial lysates supplemented with 1~2 M NaCl to induce phase separation [11]. The final material after purification yielded ~90 mg/L of V96, ~40 mg/L of S96, and ~10 mg/L of LV96 with > 98% purity, as verified by SDS-PAGE (Figure 1A). The precise determination of molecular weight by MALDI-TOF for V96, S96, and LV96 was reported previously [10,12]. To determine the *T_t_* of ELPs, optical density at 350 nm over a range of temperatures was measured (Figure 1B). All ELPs tested showed a negative correlation between the *T_t_* and the ELP concentration [13], and the phase diagram was fit by Equation 1 (Table 1).

### 3.2. ELPs Display Differential Attachment to Commercially Available Contact Lenses

**Figure 2:**
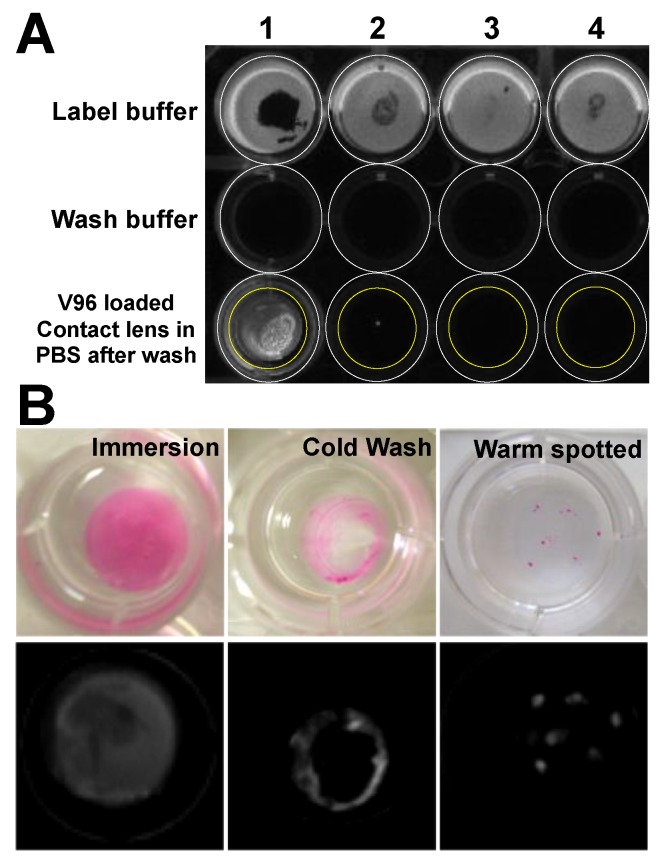
ELP selectively phase separate onto Proclear compatibles^TM^ contact lens. (**A**) Among four types of contact lenses tested, rhodamine-labeled V96 preferentially phase separated onto Proclear compatibles^TM^ contact lens. 1: Proclear compatibles^TM^; 2: Dailies AquaComfort Plus^TM^; 3: Acuvue OASYS^®^; 4: Acuvue Advance Plus^®^. Label buffer: 50 µM rhodamine-labeled V96 in PBS; Wash buffer: ddH_2_O used for gentle wash after contact lens incubation with label buffer. White circles: each well in 12-well plate; yellow circles: contact lens in the well. (**B**) Different spatial deposition patterns for rhodamine-labelled V96 on Proclear compatibles^TM^ contact lens were evaluated. The entire lens can be labeled during complete immersion in a warm solution (immersion), the central field can be depleted by a cold PBS wash (cold wash), or individual positions can be labeled by warm pipet spotting. Upper: white light; lower: fluorescence.

Discovery of ELPs’ attachment to contact lenses came from a quick screen of four commonly marketed contact lenses, including Acuvue Oasys^®^, Acuvue Advance Plus^®^, Dailies AquaComfort Plus^TM^ and Proclear Compatibles^TM^ (Table 2). Surprisingly, rhodamine-labeled V96 selectively attached to Proclear Compatibles^TM^ contact lenses at 37 °C after overnight incubation in PBS solution. This attachment remained stable at 37 °C in PBS solution for more than 24 h (Figure 2A). Motivated by the rationale that the delivery system itself should not scatter light within the central visual field, we investigated whether it was possible to arrange the ELP only around the periphery of the contact lens using a cold wash. Interestingly, by controlling the location of cold washing and warm spotting, the final deposition pattern on the lens could be controlled (Figure 2B).

### 3.3. ELP-Mediated Phase Separation Enhances Attachment to Proclear Compatibles^TM^

**Figure 3:**
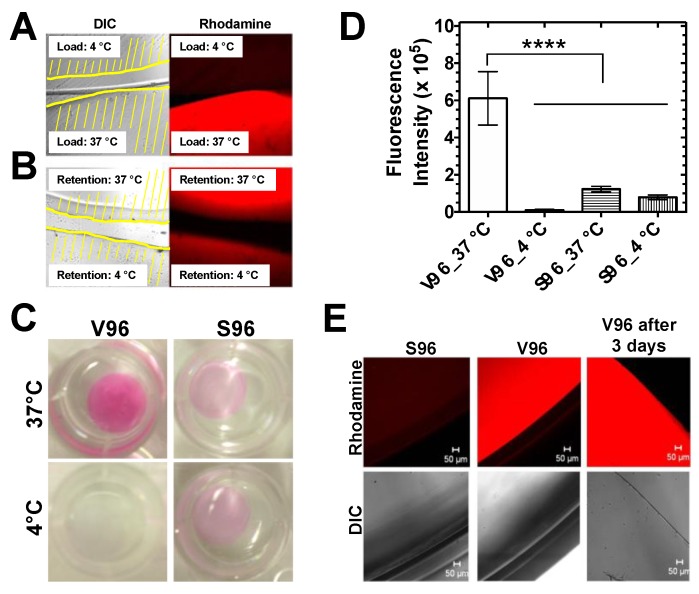
Coacervation of a temperature-responsive ELP enhances loading onto Proclear Compatibles^TM^ contact lenses. (**A**) Contact lenses were loaded overnight at 4 or 37 °C with rhodamine-labeled V96 (100 µM) and imaged side by side using confocal microscopy. Each lens’ location is depicted by yellow lines upon the differential interference contrast (DIC) channel. (**B**) A contact lens loaded with V96 (100 µM) at 37 °C was cut into halves and incubated at 4 or 37 °C in ddH_2_O overnight. Side-by-side confocal microscopy shows that the half incubated at 37 °C retains most of the V96 label. (**C**) Shown are representative pictures of lenses loaded with rhodamine-labeled V96 or S96 at 37 or 4 °C for 24 h and washed. (**D**) Total fluorescence intensity associated with lenses loaded overnight with V96 or S96 at different incubation temperature. Mean ± SD, *N* = 3, ****p < 0.0001. Significant differences between: V96_37 °C vs. V96_4 °C (*p* = 0.00004); V96_37 °C vs. S96_37 °C (*p* = 0.0002); V96_37 °C vs. S96_4 °C (*p* = 0.00009). (**E**) Confocal microscopy was used to observe lenses incubated overnight with rhodamine-labeled S96 and V96 at 37 °C and gently washed. Even after 3 days at 37 °C, the V96 remained associated with the lens. Scale bar: 50 µm.

To test whether V96 attachment is due to coacervation of V96 at 37 °C, lenses were visualized following overnight V96 incubation at 37 °C (above *T_t_*) or 4 °C (below *T_t_*). There was a striking and significant difference in V96 deposition in response to coacervation (Figure 3A). Contact lenses were then incubated with V96 at 37 °C overnight and cut into halves. The first half was incubated at 4 °C and the second half was incubated at 37 °C. Incubation at 4 °C resulted in rapid dissociation of V96, whereas V96 was retained at 37 °C (Figure 3B). To test the effects of *T_t_* and incubation temperature on ELPs’ affinity to contact lenses, V96 (*T_t_* = 29.6 °C, 100 μM, Equation 1) was compared to a heat-insensitive control S96 (*T_t_* = 60.0 °C, 100 μM, Equation 1). After 24 h incubation, total attachment of V96 at 37 °C was about 5-fold higher than that of S96 at 37 °C; and 59-fold higher than that of V96 at 4 °C and 8-fold higher than that of S96 at 4 °C (Figure 3C,D). The contact lens association with S96 at 37 °C, V96 at 4 °C, and S96 at 4 °C did not differ significantly from each other (*p* > 0.50). The difference in contact lens association between V96 and S96 at 37 °C was confirmed using confocal microscopy (Figure 3E). Heat-insensitive S96 washed away immediately prior to imaging. However, V96 coacervates decorated the lens uniformly, even after 3 days of incubation at 37 °C (Figure 3E). Although the specific biophysical interactions between ELPs and contact lenses remains to be explored, the ProClear lens composition (Table 2) clearly demonstrated both a non-specific association with S96 and a coacervate-dependent association with V96 when incubated above its transition temperature.

### 3.4. Coacervation Prolongs the Retention of ELPs on Proclear Compatibles^TM^ Contact Lenses

**Figure 4:**
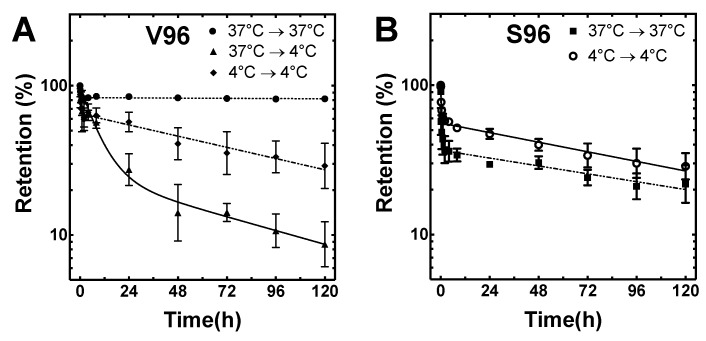
ELP retention on Proclear Compatibles^TM^ lenses depends on ELP *T_t_* and incubation temperature. (**A**) Release profiles of group 1 (V96_37 °C → 37 °C), group 2 (V96_37 °C → 4 °C), and group 3 (V96_4 °C → 4 °C) were shown. (**B**) Release profiles of group 4 (S96_37 °C → 37 °C) and group 5 (V96_4 °C → 4 °C) were shown. Small aliquots of the incubation solution were sampled over time and the fluorescence intensity of these samples were measured to estimate lens retention (Equation 3). Lines joining data points represent a best-fit to a biexponential decay model (Equation 4). Mean ± SD, *N* = 3.

Having demonstrated that ELP phase separation enhances loading of V96, the retention of ELPs was explored following washing. Five groups were evaluated for the retention of rhodamine-labeled ELPs and lenses: group 1) load V96 at 37 °C and retention at 37 °C (V96_37 °C → 37 °C); group 2) load V96 at 37 °C and retention at 4 °C (V96_37 °C → 4 °C); group 3) load V96 at 4 °C and retention at 4 °C (V96_4 °C → 4°C); group 4) load S96 at 37 °C and retention at 37 °C (S96_37 °C → 37 °C); group 5) load S96 at 4 °C and retention at 4 °C (S96_4 °C → 4 °C). After one week of lens retention testing in PBS, group 1 (V96_37 °C → 37 °C) retained ~ 80% of the initial fluorescence, that was mostly lost from all others (Figure 4A,B). Groups 3, 4, and 5 showed similar retention profiles, while group 2 lost ~ 75% of initial signal during the first 24 h. When both incubation and retention temperatures were below T_t_, little difference was observed in either total fluorescence loaded (Figure 3D) or retention (groups 3, 4, and 5). The relationship between ELP retention and coacervation is most evident by comparison of groups 1 and 2. To understand retention kinetics, we attempted to fit each dataset first by a one-phase and then by a two-phase decay model. The two-phase disassociation model was best (*p* < 0.0001) and was applied to the estimation of the terminal half-life and percentage of material lost to washing through fast release (Table 3). Most notably, the area under the curve (AUC) of group 1 during a one-week period (AUC_0-120_) was about 4-fold higher than group 2, 2-fold higher than group 3, 3-fold higher than group 4, and 2-fold higher than group 5. The extrapolated total AUC (AUC_0-Inf_) for group 1 was about 119-fold higher than group 2, 45-fold higher than group 3, 55-fold higher than group 4, and 44-fold higher for group 5.

### 3.5. Co-Incubation of LV96 with Proclear Compatibles^TM^ Enables Transfer to Cultured HCE-T Cells

**Figure 5:**
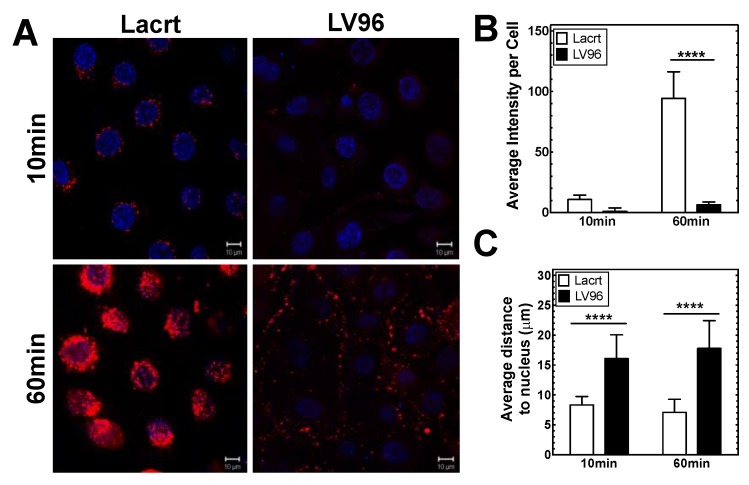
HCE-T cells associate with both recombinant Lacritin (Lacrt) and a Lacritin-V96 fusion (LV96). (**A**) Representative pictures showing live-HCE-T cell targeting and uptake of 10 µM rhodamine-labeled lacritin (Lacrt) or LV96 over 1 h at 37 °C in complete media. Red: rhodamine-labeled Lacrt or LV96; Blue: DAPI-stained nuclei. Bar = 10 µm. (**B**,**C**) Image analysis was used to quantify (**B**) integrated intensity per cell and (**C**) average distance to the nucleus of LV96 vs. Lacrt. Mean ± SD, *N* = 9 measurements, ****p < 0.0001.

**Figure 6:**
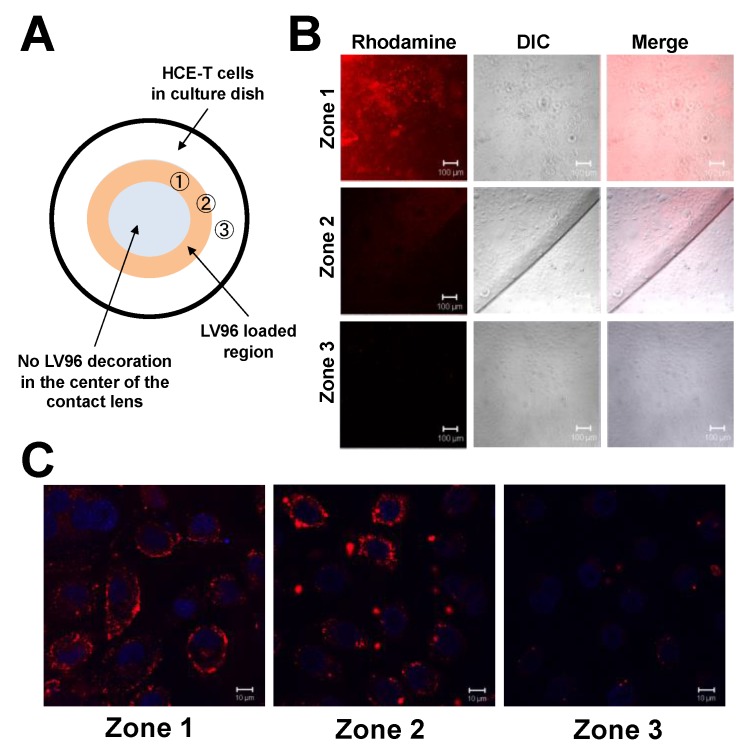
Spatial proximity is required for the efficient transfer of LV96 from Proclear Compatibles^TM^ contact lenses to cultured HCE-T cells. (**A**) Cartoon showing contact lens loaded with a ring pattern of LV96 with three zones indicated: 1) under the lens; 2) at the edge of the lens, and 3) distal to the lens. (**B**) Confocal imaging was performed to confirm the location of the rhodamine-labeled LV96 bound to a lens above cultured HCE-T cells, which were incubated for 1 h at 37 °C in complete media. Bar = 100 μm. (**C**) High magnification images show efficient association of rhodamine-labeled LV96 with cultured HCE-T cells in zones 1 and 2. In zone 3, less labeling was apparent. Red: rhodamine-labeled LV96; Blue: DAPI-stained nuclei. Bar = 10 μm. Images shown are representative from at least three independent experiments.

To explore cellular delivery from ELP-loaded contact lenses, lacritin, an abundant protein from normal human tears [14], was selected. Topical lacritin, including lacritin-ELP [12,15], promotes basal tearing and corneal wound repair in rabbit and mouse models [15] which makes it a potential treatment for dry eye disease and cornea wound healing. We first added rhodamine-labeled lacrtin-V96 (LV96) or recombinant lacritin (Lacrt) to HCE-T cells (Figure 5A). After 60 min, high levels of rhodamine-labeled lacritin had become internalized, whereas LV96 remained associated with the cell surface in lower relative amounts (Figure 5B). Accordingly, the average nuclei to closest rhodamine pixel distance was significantly greater for LV96 versus recombinant lacritin after both 10 and 60 min (Figure 5C), possibly due to steric hindrance with lacritin ligand syndecan-1 [16] on the cell surface and/or with endocytic machinery. These observations are in accordance with our previous report of comparably low cellular targeting and delay on cellular uptake of LV96 compared to Lacrt, mainly due to the fusion of V96 and its ability to coacervate at 37 °C [12]. Nonetheless, evidence of LV96 cell targeting was clearly apparent. We next tested delivery from LV96-decorated contact lenses in which rhodamine-labeled LV96 was restricted to a peripheral ring. HCE-T cells growing directly under (zone 1), adjacent (zone 2) or outside (zone 3) the ring were scrutinized after one hour (Figure 6A). Most zone 1 cells were covered with LV96, versus progressively less coverage of zones 2 and 3 cells (Figure 6B) with zone 3 showing negligible targeting and uptake (Figure 6C).

## 4. Discussion

Drugs delivered by drops on the eye can suffer from an inefficient pharmacokinetic profile beginning with an initial transient overdose, followed by a prolonged period of drug insufficiency [17], further diminished by blinking, reflex tearing, and nasolacrimal system drainage. Only 1~7% by volume generally targets the eye [18]. Emerging drug delivery systems include: ophthalmic ointments, viscous polymer vehicles, nanoparticles, in situ gel-forming systems, iontophoresis, and modified punctal plugs [19,20,21]. Problems include the lack of optically transparency, instability, difficulty inserting and discomfort [22]. Druggable contact lenses offer an attractive alternative [23] as they are conceptually simple, and should not impair vision [24]. Strategies include simple immersion in drug [25,26], inclusion of drug-loaded colloidal nanoparticles [27,28] and molecular imprinting [29] with a focus on small molecule therapeutics, including cyclosporine A [30], timolol [31,32] and Latanoprost [33]. Also, adipose-derived stem-cells loaded contact lens were tested for the treatment of acute alkaline burns [34]. In general, none have succeeded in exerting full spatiotemporal control over drug delivery towards eliminating a drug bolus on application, and consequential side effects. Further, none have successfully developed a method to slowly deliver protein therapeutics [35,36], which is more challenging due to steric hindrance and complex template design. ELP fusion proteins offer a solution, and custom contact lenses are not required. We report here the selective adsorption of thermo-responsive ELPs to a commercially available contact lens, elucidate the *T_t_* dependence for attachment and detachment, and we further showed proof of the concept by the spatiotemporal delivery of model ocular protein drug lacritin via contact lens to HCE-T cells. Although ELP-contact lens delivery systems have not met the desirable zero-order release, 80% retention (group 1: V96_37 °C → 37 °C) after initial release may provide a way to maintain therapeutic dose for an extended time on the ocular surface that can overcome short half-lives, low tear bioavailability, and prolonged sub-therapeutic concentrations accompanying eye drop instillation. In addition, by changing the ELP composition and raising its transition temperature closer to that at the ocular surface, future studies may show that it is possible to titrate the rate of release necessary for optimal therapy.

The core questions that have to be answered would be the mechanism behind ELP-contact lens adsorption that is uniquely observed with Proclear Compatibles^TM^ and further stabilization of ELP adsorption upon coacervation. As can be seen from the S96 association with the lenses, even below their transition temperature (*T_t_*), ELPs nonspecifically adsorb to ProClear contact lenses. Upon an increase in temperature, we hypothesize that adsorbed ELPs nucleate coacervation, which recruits additional ELPs from solution. The exact interaction that leads to the adsorption of ELPs to ProClear remains unknown. Electrostatic interactions are ruled out because neither the ELP nor the ProClear formulation contain excess charged groups. Van-der Waals interactions are possible between the hydrophobic moiety on the abundant Valine residues on the ELP and hydrophobic groups on the ProClear formulation. A third possibility is that hydrogen bonding may play an important role for this adsorption. Two representative biopolymer modalities used to study temperature-dependent phase transition behavior are ELPs and Poly(N-isopropylacrylamide) (PNIPAM) [37]. Both polymers undergo coacervation above their transition temperature (*T_t_*). During coacervation, highly unordered PNIPAM remains unordered with negligible amount of additional hydrogen bond formation [38]; however, highly unordered ELPs are thought to form more ordered structure (type II β-turns, β-spirals, or distorted β-sheets) that nucleate the growth of coacervate phases [39]. This process involves the formation of a hydrogen bond called ‘1–4 hydrogen bond’ (C=O of the first residue (valine) and the NH of the fourth residue (guest residue)) within the ELP pentapeptide [40]. We propose that these abundant hydrogen bonds may participate in hydrogen bonding with organic phosphates on the phosphorylcholine (PC)-coated contact lens [41]. Given that every ELP pentamer forms one additional hydrogen bond upon coacervation, and each ELP contains 96 pentameric units, even small contributions from these hydrogen bonds to phosphate-mediated hydrogen bonding between ELP coacervates and contact lens may promote ELP adsorption and coacervation. It should be emphasized that the adsorption of V96 coacervates to the contact lens does not indicate this particular brand is sub-optimal in preventing protein adsorption because ELPs and their coacervates do not have the amino acid compositions, biophysical properties, or affinity for typical proteins [42]. Given that only Proclear Compatibles^TM^ contains phosphorylcholine on the contact lens surface among those tested, further investigations comparing the adsorption of ELP coacervates to pHEMA vs. pHEMA+PC may give more insights towards the molecular bonding that links ELPs to this formulation of lenses. 

Several aspects have to be considered when using ELP coacervates on the lens for future therapeutic purposes. First, the presence of the ELP layer may alter O_2_ permeability which may lead to insufficient oxygen supply to the cornea surface. Second, ELP coacervates may affect visual acuity. These possible limitations can be solved by loading ELP coacervates in a defined region, e.g., on the edge of the ring (Figure 2 and Figure 6). This may guarantee sufficient oxygen supply to the cornea surface with clear vision, while ELP drugs diffuse into the cornea surface.

The delivery of therapeutic ELP fusion proteins to the ocular system and its compatibility, biodistribution, or therapeutic efficacy were studied by us and other groups. Examples include the delivery of intravitreal αB crystallin ELP fusion to a mouse model of age-related macular degeneration [43], topical lacritin-ELP as an eyedrop for healing of mouse corneal wound model [44], intravitreal injection of poly(VPAVG) particles to a normal rabbit model [45], and cell-penetrating peptide ELP fusions to a normal rabbit model [46]. As these modalities have shown ELPs as a highly promising ocular drug delivery platform, it is necessary to have a unique set of evidence that the delivery of ELPs or ELP fusions can be rerouted to the ocular system via contact lenses in a more hassle-free fashion. 

Different from other reported contact lens-mediated drug delivery systems, our study represents a ‘drug refillable system,’ which would enable refill of a drug at home, by the patient [47,48]. Given the panel of ELP fusion modalities developed in our laboratory, the data presented in this study indicate that this system’s application catalogue can be broadened to anti-inflammation agents, antibiotics, polypeptides and diverse protein/antibody therapeutic libraries via encapsulation or recombinant protein expression strategies. While its underlying mechanism remains to be elucidated, our discovery may provide a promising new avenue to circumvent challenges associated with the effective delivery of therapeutics to the ocular surface.

## Figures and Tables

**Table 1:** Summary of the elastin-like polypeptides (ELPs) involved in this study.

Label	Amino Acid Composition	*MW (kDa)	*T_t_* (°C) at 25 μM	Phase Diagram	
				Slope, *m* [°C/log_10_(μM)]	y-intercept, *b* [°C]
S96	G(VPGSG)_96_Y	38.4	57.6	−1.669	59.31
V96	G(VPGVG)_96_Y	39.5	31.6	−3.252	36.06
LV96	**Lacritin-G(VPGVG)_96_Y	52.3	26.8	−1.192	28.56

*MW determined by MALDI-TOF analysis. **Lacritin (12.7 kDa) amino acid sequence: EDASSDSTGADPAQEAGTSKPNEEISGPAEPASPPETTTTAQETSAAAVQGTAKVTSSRQELNPLKSIVEKSILLTEQALAKAGKGMHGGVPGGKQFIENGSEFAQKLLKKFSLLKPWA.

**Table 2:** Summary of the contact lenses involved in this study.

Brand Name	Manufacturer	Polymer	Monomer	ELP Attachment
Proclear Compatibles^TM^	CooperVision	Omafilcon A	pHEMA/PC	+
Dailies AquaComfort Plus^TM^	CIBA Vision	Nelfilcon A	HPMC/PEG/PVA	−
Acuvue Oasys^®^	Johnson & Johnson	Senofilcon A	pHEMA + DMA + mPDMS + siloxane macromer + TEGDMA + PVP	−
Acuvue Advanced Plus^®^	Johnson & Johnson	Galyfilcon A	pHEMA + DMA + mPDMS + siloxane macromer + EGDMA + PVP	−

pHEMA: poly(hydroxyethyl methacrylate); HPMC: Hydroxypropyl methylcellulose; PC: phosphorylcholine; mPDMS: monofunctional poly(dimethylsiloxane); DMA: N,N-dimethylacrylamide; EGDMA: ethyleneglycol dimethacrylate; TEGDMA: tetraethyleneglycol dimethacrylate; PVP: poly(vinyl pyrrolidone); PVA: poly(vinyl alcohol); PEG: poly(ethylene glycol).

**Table 3:** Release kinetics of ELPs from Proclear Compatibles^TM^ contact lenses.

Parameters	Group 1	Group 2	Group 3	Group 4	Group 5
ELP	V96	V96	V96	S96	S96
Label Temp (°C)	37	37	4	37	4
Release Temp (°C)	37	4	4	37	4
Percent Fast (%)	16.8 (15.6~18.0)	75.0 (63.7~86.2)	35.1 (27.9~42.2)	63.3 (59.3~67.3)	55.9 (52.1~59.6)
*k_fast_* (h^−1^)	2.9 (2.0~3.9)	0.1 (0.06~0.2)	3.3 (0.0~6.7)	3.4 (2.0~4.7)	2.4 (1.4~3.7)
*t_1/2,fast_* (h)	0.2 (0.18~0.35)	5.8 (4.0~10.9)	0.2 (0.1~inf.)	0.2 (0.1~0.3)	0.3 (0.2~0.5)
*k_slow_* (h^−1^)	0.0002 (0.0~0.0004)	0.009 (0.004~0.01)	0.007 (0.005~0.009)	0.005 (0.003~0.007)	0.006 (0.005~0.007)
*t_1/2,slow_* (h)	4615 (1815~inf.)	78.3 (49.8~183.7)	96.2 (76.9~128.4)	137.1 (101.6~210.7)	112.5 (95.9~136.0)
AUC_0-120h_	9938	2565	5156	3245	4660
AUC_0-Inf_	418564	3525	9302	7608	9436
*R^2^*	0.89	0.88	0.81	0.82	0.93

*Fast* and *slow* represent the fast and slow exponential decay phase in the two-phase dissociation (decay) model, respectively. Values indicate the mean (95% CI).
